# Effectiveness of a combination prevention strategy for HIV risk reduction with men who have sex with men in Central America: a mid-term evaluation

**DOI:** 10.1186/1471-2458-14-1244

**Published:** 2014-12-04

**Authors:** Rebecca Firestone, Jorge Rivas, Susana Lungo, Alejandra Cabrera, Susan Ruether, Jennifer Wheeler, Lung Vu

**Affiliations:** Population Services International, 1120 19th Street, NW, Suite 600, Washington, DC 20036 USA; Pan-American Social Marketing Organization (PASMO), 13 Calle 3-40, Zona 10, Edificio Atlantis, Nivel 13, Oficina 1305, Guatemala City, Guatemala; Population Council, 4301 Connecticut Ave NW, Suite 280, Washington, DC 20008 USA

**Keywords:** Men who have sex with men, Combination prevention, Central America, Condom use, HIV testing and counseling, HIV prevention interventions, Social marketing

## Abstract

**Background:**

Despite over a decade of research and programming, little evidence is available on effective strategies to reduce HIV risks among Central American men who have sex with men (MSM). The Pan-American Social Marketing Organization (PASMO) and partners are implementing a HIV Combination Prevention Program to provide key populations with an essential package of prevention interventions and services: 1) behavioral, including interpersonal communications, and online outreach; 2) biomedical services including HIV testing and counseling and screening for STIs; and 3) complementary support, including legal support and treatment for substance abuse. Two years into implementation, we evaluated this program’s effectiveness for MSM by testing whether exposure to any or a combination of program components could reduce HIV risks.

**Methods:**

PASMO surveyed MSM in 10 cities across Guatemala, El Salvador, Nicaragua, Costa Rica, and Panama in 2012 using respondent-driven sampling. We used coarsened exact matching to create statistically equivalent groups of men exposed and non-exposed to the program, matching on education, measures of social interaction, and exposure to other HIV prevention programs. We estimated average treatment effects of each component and all combined to assess HIV testing and condom use outcomes, using multivariable logistic regression. We also linked survey data to routine service data to assess program coverage.

**Results:**

Exposure to any program component was 32% in the study area (n = 3531). Only 2.8% of men received all components. Men exposed to both behavioral and biomedical components were more likely to use condoms and lubricant at last sex (AOR 3.05, 95% CI 1.08, 8.64), and those exposed to behavioral interventions were more likely to have tested for HIV in the past year (AOR 1.76, 95% CI 1.01, 3.10).

**Conclusions:**

PASMO’s strategies to reach MSM with HIV prevention programming are still achieving low levels of population coverage, and few men are receiving the complete essential package. However, those reached are able to practice HIV prevention. Combination prevention is a promising approach in Central America, requiring expansion in coverage and intensity.

## Background

Despite several decades of work [1, 2], there is still insufficient evidence of how to effectively reach men who have sex with men (MSM) in low and middle-income countries with effective prevention programming, particularly in settings where stigma and homophobia are prevalent and MSM are difficult to reach. An estimated 112,000 people in Central America were newly infected with HIV in 2010. MSM in Central America are one of the most affected key populations in the region, with HIV prevalence ranging from 7.5% to 11.1% [3, 4]. Investments have been made in Central America to target key populations including MSM, but evidence is extremely limited on the effectiveness of these investments [5–7]. We aim here to assess the effectiveness of one combination prevention program for reducing HIV risks among MSM across 5 countries in Central America.

That MSM in Central America experience elevated HIV risks is well documented. High levels of unprotected anal intercourse have been reported in Nicaragua and El Salvador. Condom use is frequent, but the picture of consistent condom use is not clear, and in 2013, 60.2% of MSM reported sometimes or never using condoms with their partners [8, 9]. Use of HIV testing and counseling (HTC) is variable across the region, despite high levels of awareness of services [8–10]. HIV testing in the past year ranged from 54% in San Salvador, El Salvador in 2008 to 72% in Chinandega, Nicaragua in 2009 [11, 12].

HIV risk behaviors among MSM occur in a context of stigma and violence. Central America experiences high levels of generalized violence along with homophobia targeted against MSM and transgender women [13–15]. Stigmatizing social environments increase the vulnerability of MSM to a range of physical and mental health risks [1, 8, 16]. Violence against men, especially homosexual men, including forced sex, is common, along with MSM being refused services from health care providers [8, 13, 16, 17].

Given structural factors, such as homophobia and violence, it is difficult to reach targeted populations at impactful levels of coverage and challenging to maintain these contacts. In Central America and elsewhere, the HIV community is increasingly mobilized around combination prevention strategies, recognizing that it is not possible to get traction on the epidemic through isolated efforts that do not address contexts of risk [18, 19]. A combination prevention approach includes biomedical interventions that block the biological mechanisms through which HIV transmission occurs or reduce the chance of acquisition of infection, such as provision of condoms, STI treatment, pre-exposure prophylaxis, and post-exposure prophylaxis, as well as evidence-based behavioral interventions that use communications channels to promote behaviors that reduce HIV risk – which on their own can be effective but are difficult to sustain - as well as generate demand for biomedical interventions [20, 21]. Critically, combination prevention also includes structural interventions to address the social drivers of the epidemic to create environments more supportive for vulnerable populations [18, 20–22]. Structural interventions cover a wide scope of approaches, for example cash transfers, community dialogue, sensitivity training for law enforcement and health providers, and political advocacy, to address the multiple levels of social interactions that influence HIV risk and transmission [22]. These interventions operate at the population level and aim to address an individual’s risk through indirect, upstream mechanisms that shape norms, behaviors and health outcomes in the population as a whole [23]. Best practice recommends that the specific combination of interventions to be implemented from within the three components should be designed based on local epidemiology and social conditions [24].

While combination prevention is increasingly the consensus framework for HIV prevention, there is as yet little evidence on the effectiveness of different combination prevention strategies [25]. The need to evaluate the combination prevention approach is clear, given the complexity of evaluating multi-level programming, but few dedicated studies have been conducted [26].

### Program description

With support from USAID (United States Agency for International Development), the Pan-American Social Marketing Organization (PASMO) and Population Services International (PSI)/Mexico began implementing a Combination Prevention Program for HIV with community-based partners in 2011 across Belize, Guatemala, El Salvador, Nicaragua, Panama, Costa Rica, and Mexico. The program is designed to increase access to HIV prevention interventions in order to reduce risk behaviors among key populations and reduce social hostility that contributes to stigma and discrimination.

PASMO has defined an essential package of interventions for MSM following a combination prevention approach [27]. To receive a complete package, an individual client should have a) participated in at least three behavior change communication interventions conducted by outreach teams or through online outreach, b) received a referral for biomedical services such as HTC or screening for sexually transmitted infections (STI), and c) received a referral to a set of complementary services.

The behavioral component includes integrated behavior change communications activities conducted by outreach workers. Based on the transtheoretical model and Population Services International’s PERForM framework, these activities were designed to generate demand for the program’s products and services, while also encouraging individuals reached to assess their health risks and build skills in HIV prevention [28–30]. Activities are conducted in person one-on-one, one-to-group or online through cyber-educators. Using Prochaska’s Stages of Change framework, outreach workers identify the stage a targeted individual is in to be able to practice a specific behavior being promoted, such as condom use or use of HTC, and tailor delivery of activities according to that stage through discussion and reflection [29]. Outreach workers also ensure that condoms and lubricants are available for sample or purchase near to where activities are being implemented [31].

The biomedical component is core to the Combination Prevention program’s design. Services provided include HTC, STI screening and treatment, and for people living with HIV, referrals for care and treatment, including antiretroviral therapy. A variety of service providers participate in the program, including private laboratories, private and NGO clinics, and some public sector facilities. HTC is provided through fixed and mobile services, according to where the program is operating [31].

The addition of complementary services was designed to support program clients to locate resources that help them negotiate potential stigma and discrimination [31]. These specifically include referrals to designated service providers for support groups, drug and alcohol treatment, legal support, and violence prevention services. In addition, the program operates beyond the interpersonal level to address stigma and discrimination and change social norms through sensitization and training of health care providers to improve perceived quality of care, media training, and mobilization of civil society leaders to foster public dialogue on homophobia and discrimination against people living with HIV.

Integrated HIV prevention teams conduct behavioral interventions and referrals to biomedical and complementary services in identified high-risk zones in program cities. For MSM, these zones are areas with large concentrations of nightclubs, bars, parks and areas where community organizations have identified that MSM are likely to meet each other. Teams include outreach workers, PASMO sales staff responsible for ensuring availability and access to condoms, community leaders, and service providers. When clients are contacted by an outreach worker, they receive an in-person behavioral intervention plus a packet of vouchers for other behavioral interventions, condoms, biomedical services, and complementary services. The program tracks client contacts and use of referral vouchers via unique identifier codes assigned to each client contacted [32]. While conducting direct service delivery and coordinating with other program partners, PASMO and partners have also implemented a set of advocacy strategies as part of the structural component of the program. Advocacy is designed to encourage public discussion of HIV transmission and encourage reduction in social hostility towards key populations including MSM [33].

We aimed in this paper to provide early results on PASMO’s combination prevention program, as targeted to MSM in Central America. Two years into implementing the program, our objectives were to 1) assess population-level coverage of specific and combined intervention components and 2) determine whether program exposure to any or a combination of components was associated with HIV risk reduction behaviors, specifically condom and water-based lubricant use, HIV testing and counseling, and appropriate treatment of STIs.

## Methods

### Data sources and measures

We used behavioral survey data and data extracted from PASMO’s program monitoring system for this analysis.

#### Regional survey of MSM

From September-November 2012, PASMO recruited MSM from 10 cities in six Central American countries into a survey using respondent-driven sampling (n = 4231). The survey was conducted in areas where the Combination Prevention program operates: Belize City in Belize, San Jose in Costa Rica, San Salvador and Santa Ana in El Salvador, Guatemala City and Quetzaltenango in Guatemala, Managua and Chinandega in Nicaragua, and Panama City and Colón in Panama. These cities were selected because they had on-going program operations and national level evidence indicated high projected HIV prevalence in each city [3, 34]. Men were eligible to participate in the study if they were aged 18–40, had had anal sex with another man in the past 3 months, were resident in the study city, and knew the name or alias of the person who had recruited that respondent to participate. Respondent-driven sampling is a chain referral sampling technique developed to improve the population representativeness of surveys of hard-to-reach populations [35, 36]. We recruited 1–3 seeds in each city. We selected seeds based on socio-economic status, self-identification of sexual orientation, and working in commercial sex. Each seed was asked to recruit three peers within his social network to participate in the study, and subsequent study participants were asked to recruit an additional three peers.

Data was collected through in-person interviews administered by a male enumerator in a location that ensured the respondent’s privacy. The survey instrument included questions on socio-demographic characteristics, sexual behaviors, STI symptoms, use of STI treatment and HTC services, mass media and program exposure, knowledge and attitudes regarding HIV prevention, and experience with violence. The PSI Research Ethics Board provided ethical approval of this survey along with Ministries of Health in Belize, El Salvador, Guatemala and Nicaragua. The Panama National HIV/AIDS Program and the University of Medical Sciences in Costa Rica approved the study as well. Individuals were asked to provide informed consent before participating in the interview. No identifying information was collected from respondents.

For this analysis, we did not use data from Belize because program operations were not completely comparable. We also excluded transgender women (n = 400 self-identified cases) from this analysis because transgender women are increasingly considered to be a separate population programmatically and epidemiologically [37]. Our final analytical sample was 3531 men.

#### Routine data

We also used data extracted from PASMO’s routine program monitoring system. PASMO’s system for applied monitoring (SAM) tracks BCC activities and service utilization across all program implementers in six countries. A key feature of the SAM is the use of unique identifier codes (UICs) to track individual clients across program contacts. Unique identifier codes are based on personal information that does not vary over time and that clients can easily recall. Monitoring data are collected on BCC activities by type of activity and target population reached, messages and behaviors, referrals initiated and completed, plus client use of HTC, and STI treatment, and any referrals to other services, such as CD4 count and viral load testing for people living with HIV. Data on program contacts and service utilization are aggregated by country and reported to partners on a monthly basis. For this analysis, we extracted data from October 1, 2011-September 30, 2012. No identifying information on program clients was collected, and analysis of this data was determined to not constitute research with human subjects. From extraction of routine data, we identified 15031 program contacts across the five countries.

### Measures

#### Outcome measures

Five outcome measures were used for this analysis, all taken from behavioral survey data: 1) condom use at last sex, 2) condom use with water-based lubricant at last sex, 3) consistent condom use in the past 30 days, 4) receipt of an HIV test in the past 12 months, and 5) seeking medical care for a suspected STI symptom in the past 12 months. Consistent condom use was defined as using a condom from start to finish for all sex acts with any partner type for the past 30 days. We measured consistent condom use by partner type for commercial, casual and regular partners. Commercial partners were partners from whom the respondent received money or to whom the respondent gave money for sex. Casual partners were defined as partners with whom the respondent had sex with no emotional attachment and no financial transaction. Regular partners were defined as partners with whom the respondent had engaged in sex for more than 3 months and felt connected to, either by love or friendship.

#### Program exposure

We created several measures of program exposure from behavioral survey data. These measures were designed to capture specific components of the combination prevention approach in order to capture program operations and assess potentially additive effects of the different components. Exposure to behavioral interventions was defined as having been contacted by an outreach worker representing the project. Exposure to biomedical interventions was defined as having received a voucher for HIV testing and counseling, to capture the referral process. Exposure to complementary interventions was defined as receiving a voucher for other services. We defined any exposure to the program as receipt of any one of these components. We also created a measure of having received all three components, defined as the combination package.

After extracting individual records from the program monitoring system, we created measures of receipt of behavioral interventions (y/n, and number of interventions), receipt of biomedical services (y/n and number of services), and receipt of complementary interventions (y/n and number of services) for this analysis.

#### Other measures

We considered a range of other factors from the survey data for this analysis. We looked at socio-demographic factors of age, education (primary education or less, some secondary education, any higher education), and an index of socio-economic status. The index of socio-economic status was constructed following methods of the Mexican Market Research Association and grouped individuals into categories of high, medium and low socio-economic status [38]. We also considered how men described their sexual identity (gay, bisexual, heterosexual).

We considered a series of factors assessing men’s social interactions with gay, transgender and other MSM communities. These included: friends’ knowing the respondent’s sexual orientation, having participated in a gay/transgender event in the past 12 months, and going to a gay bar/disco in the past 30 days. We also considered exposure to other HIV/AIDS programming targeting MSM, measured as whether the respondent received a condom for free in the past 12 months. All factors were treated as dichotomous variables.

### Analysis of survey data

#### Weighting

For descriptive sample characteristics, we present un-weighted data. We conducted weighted analyses to assess program effects using multiple logistic regression. Because of our sampling and analysis strategy, we calculated weights using pooled data. Several types of weights were calculated and adjusted for in regression models. First, we calculated RDS weights using RDSAT software, which accounts for network size and recruitment patterns for each city [39]. Second, we estimated city weights to account for differential proportions of the sample and estimated population size of each city. Because reliable estimates of the population size of men who have sex with men in all 9 cities were not available, we weighted by the city population of men 15–39 years [40]. Population size estimates were retrieved from national statistics bureaus in each country [41–45]. Age- and sex-specific population size estimates were not available for Guatemala, and we therefore applied the national proportion of men in Guatemala to the age distribution for each city. A third set of weights was used as well, derived from the matching procedure described below. These weights were multiplied together and applied in regression models. Data analysis was conducted in Stata 13 and SPSS 22.

#### Statistical matching

We next used a statistical matching technique, coarsened exact matching (CEM), to improve the quality of our estimates of program effects [46–48]. Coarsened exact matching was used to create statistically equivalent groups of survey respondents exposed and not exposed to any program component. This quasi-experimental approach allowed us to designate a counterfactual (no exposure to any Combination Prevention program components) when an experimental design was not feasible, since program implementation was ongoing at scale across several countries and was not designed or implemented with intervention and control groups [47, 49]. Coarsened exact matching is a monotonic imbalance matching method designed to reduce imbalance between treatment and control groups in observational data [49, 50]. CEM assigns each case into one of a specified set of strata in which members are exactly matched on a set of coarsened, or categorized, variables [46]. Matched cases are then assigned a weight specific to that stratum and representative of the proportion of all cases present in the stratum [51, 52].

We matched survey respondents on factors likely to influence exposure to PASMO’s combination prevention program, given the strong likelihood of selection bias in a setting where MSM are highly stigmatized [53]. We matched on education (categorized as primary education or less, any secondary education, any higher education), sexual orientation (heterosexual, homosexual, bisexual), whether the respondent’s friends knew he had sex with other men, whether the respondent had participated in a gay/transgender event in the past 12 months, whether the respondent had been to a gay bar or disco in the past 30 days, and whether the respondent had received free condoms in the past 12 months. We assessed relationships between potential matching variables and any program exposure using Spearman correlation coefficients. We matched variables that had a statistically significant correlation with program exposure at p < 0.05. An exact match made on the coarsened variables yielded an L1 measure of 4.814e^-16^, indicating minimal imbalance between respondents exposed and not exposed [46].

#### Modeling strategy

We calculated un-weighted descriptive statistics for key measures using country data and pooled data. Descriptive statistics in the pooled dataset were calculated for the full and the matched samples. We next compared distributions of program exposure in the survey data to program exposure from monitoring data by country. We then used weighted multivariable logistic regression to estimate odds ratios of average treatment effects for each measure of program participation, applying all three types of weights (RDSAT weights, city weights, CEM weights). We first estimated unadjusted odds ratios for each outcome of interest. We next adjusted for age and socio-economic status – factors not included in the matching procedure. We also investigated the effects of receiving two combination prevention channels (behavioral + biomedical and behavioral + complementary), with and without controls. The same modeling strategy was used in the full sample and with the matched sub-sample to estimate how estimates of treatment effects changed following matching.

### Analysis of combined survey and MIS data

Finally, we created a new sub-sample of cases where we could link unique identifiers between routine data and survey data. Because of the limited sample size for these linked cases, we did not apply any sampling weights, and we did not match because all cases were exposed. We conducted chi-square tests of association between type of program exposure and the outcomes of interest by country.

## Results

More than 80% of respondents across all countries were under 30 (Table 1). Half of the surveyed men were educated at the secondary level and 40% had some university, except for Costa Rica where a quarter of the sample had only primary education. The majority of men were of middle or lower socio-economic status. Half of respondents identified as homosexual or gay, 40.5% as bisexual, and 8.4% as heterosexual. Trends in sexual identity varied by country; Panama had a relatively greater proportion of men indicating they were gay/homosexual while Costa Rica and El Salvador had larger proportions of men reporting they were heterosexual. The majority (70%) of respondents indicated that they had disclosed their sexual identity to their friends, 33% had participated in a gay/transgender event in past 12 months, and 63% had visited a gay bar or disco in the past 30 days.

**Table 1:** **Characteristics of men who have sex with men in Central America, 2012**

	Costa Rica (n = 736)	El Salvador (n = 630)	Guatemala (n = 750)	Nicaragua (n = 618)	Panama (n = 797)	Pooled sample (n = 3531)	Matched sample (n = 2922)
**Socio-demographic characteristics**							
**Age**							
18–24	50.7	67.5	57.7	74.4	48.6	58.9	63.9
25–29	24.7	16.7	18.0	16.0	25.8	20.6	16.7
30–34	14.7	9.8	11.3	7.3	14.6	11.8	11.2
≥34	9.9	6.0	12.9	2.3	11.0	8.8	8.2
**Education**							
Primary or less	25.1	6.5	16.1	9.9	2.1	12.0	8.1
Secondary	57.7	22.9	23.5	72.0	68.6	49.2	44.6
Tertiary	17.1	70.6	60.4	18.1	29.2	38.8	47.3
**Socio-economic status**							
Low	54.4	57.7	39.7	70.1	60.3	56	54.7
Middle	40.7	39	50	28.7	37.3	39.5	40.2
High	4.9	3.4	10.3	1.2	2.3	4.5	5.1
**Sexual identity**							
Heterosexual	13.3	15.7	5.1	4.7	3.9	8.4	4.1
Bisexual	51.5	38.1	51.9	39.3	22.5	40.5	36.9
Gay/homosexual	35.2	46.2	43.1	56.0	73.7	51.1	59.1
**Social interaction**							
Out with friends	63.0	70.4	66.3	72.8	78.3	70.2	78.5
Participated in gay/LGBT event in past 12 months	29.3	32.5	30.4	43.4	30.8	32.9	43.8
Visited gay bar/disco in past 30 days	64.5	46.2	62.4	62.0	78.8	62.5	72.4
Received free condoms in past 12 months	47.6	63.7	78.1	67.9	40.1	58.8	71.4
Ever participated as an HIV/AIDS educator	7.5	8.7	9.5	15.2	11.4	10.4	11.7
**Behavioral outcomes**							
Condom use at last sex	84.1	84.1	90.4	84.9	82.9	85.0	84.0
Condom and lubricant use at last sex	45.3	33.9	59.5	37.0	61.3	47.5	48.7
Consistent condom use, all partners	79.3	80.4	82.1	86.4	84.4	82.2	80.4
Consistent condom use, regular partners	80.3 (n = 390)	74.2 (n = 387)	83.9 (n = 472)	86.4 (n = 391)	85.1 (n = 571)	82.3 (n = 2211)	78.9 (n = 265)
Consistent condom use, commercial partners	87.8 (n = 237)	96.5 (n = 113)	92.7 (n = 205)	95.6 (n = 91)	92.4 (n = 118)	92.0 (n = 764)	92.8 (n = 91)
Consistent condom use, casual partners	88.7 (n = 327)	90.4 (n = 230)	90.2 (n = 418)	91.3 (n = 208)	90.3 (n = 422)	90.1 (n = 1605)	91.1 (n = 188)
Received an HIV test, past 12 months	39.3	57.6	52.4	55.4	56.3	52.1	61.9
Sought medical treatment for STI episode, past 12 months	74.2 (n = 58)	75.0 (n = 88)	68.6 (n = 137)	65.9 (n = 44)	75.9 (n = 170)	72.4 (n = 497)	78.2 (n = 51)

Of the outcomes of interest, 85% of men reported having used a condom at last sex, and 48% used a condom with water-based lubricant at last sex. Regarding consistent condom use, 82% reported consistent condom use in the past 30 days with all partners. Consistent condom use was substantially higher when respondents reported having casual partners (90%) and commercial partners (92%). Just over half of respondents reported having had an HIV test in the last 12 months (52%), and of those with STI symptoms, 76% sought medical treatment.

Almost a third (32.2%) of respondents reported exposure to any component of the Combination Prevention program (Table 2). Across the five countries, exposure to the behavioral component reached 17.4% and was highest in Guatemala (27.9% overall and 74.2% of those with any exposure) and lowest in Costa Rica and Panama. Coverage of the biomedical component reached 16.4% of men regionally, with El Salvador and Panama achieving the greatest levels of biomedical exposure. Exposure to complementary interventions was reported by 11.2% of respondents regionally; the reach of the complementary component was minimal in Costa Rica but reached 65% of those with any exposure in El Salvador. Only 2.8% of respondents received all three components of the program, with El Salvador (6.1%) and Guatemala (5.5%) achieving the greatest levels of population-based coverage of combination prevention exposure.

**Table 2:** **Exposure to combination prevention intervention channels among men who have sex with men**

	***Costa Rica***			***El Salvador***			***Guatemala***			***Nicaragua***			***Panama***			***Pooled sample***	***Matched sample***
	Survey data	Component-specific % of any exposure	SAM	Survey data	Component-specific % of any exposure	SAM	Survey data	Component-specific % of any exposure	SAM	Survey data	Component-specific % of any exposure	SAM	Survey data	Component-specific % of any exposure	SAM		
N	736		1065	630		2881	750		2907	618		3886	797		4312	3531	2922
Any	18.9			41.4			40.2			37.8			25.8			32.2	35.5
Behavioral	9.5	58.8	100	23.3	56.5	91.0	27.9	74.2	84.0	19.4	56.5	97.9	9.7	50.0	98.2	17.4	18.9
Biomedical	10.8	47.1	0.0	22.7	45.7	16.5	17.5	38.7	22.7	20.3	47.8	8.2	12.1	50.0	2.8	16.4	17.2
Structural	1.8	11.8	1.2	26	65.2	3.4	16.6	45.2	0.6	5.9	17.4	3.4	4.1	18.2	1.3	11.2	12.4
Combination	0.3	0.0		6.1	15.2		5.5	16.1		1.6	4.3		0.6	4.5		2.8	3.0

SAM = system for applied monitoring.

We also report trends in program exposure from program monitoring data. Based on how the data were collected, exposure to behavioral interventions was considerably greater than to any other component across all countries, ranging from 84 to 100% of reported contacts. Only El Salvador and Guatemala reported anything other than minimal levels of biomedical contacts (16.5% and 22.7%, respectively), while El Salvador and Nicaragua had the greatest reported contacts with complementary services (both at 3.4%).

Table 3 reports findings from models estimating program effects for the outcomes of interest. We do not show modeling results for consistent condom use with casual partners or for STI treatment seeking, where there were no findings. Men exposed to any programmatic component were more likely to use condoms consistently with regular partners (OR 1.69; 95% CI 1.09, 2.62) and to have been tested for HIV (AOR 2.98; 95% CI 1.82, 4.87). Men exposed to the behavioral component were more likely to use a condom with water-based lubricant the last time they had sex (OR 1.84; 95% CI 1.08, 3.14), to use condoms consistently with regular partners (AOR 1.88; 95% CI 1.09, 3.25), and to have tested for HIV in the past 12 months (OR 1.76; 95% CI 1.001, 3.10). Exposure to the complementary component was also associated with HIV testing (OR 1.97; 95% CI 1.0, 4.05). The odds of condom and water-based lubricant use were greater when men received both the behavioral and biomedical component (OR 3.05; 96% CI 1.08, 8.64). Exposure to both behavioral and complementary components was associated with consistent condom use with commercial partners in the full sample prior to matching (OR 2.47; 95% CI 1.00, 6.09), but after matching, these results were not statistically significant. Overall, we found no evidence that receipt of all three program components was associated with any of the outcomes of interest, although this measure of exposure was rare.

**Table 3:** **Associations with combination prevention program exposure for behavioral outcomes among MSM across five Central American countries**

	Full sample (n = 3531)*		Matched sample (n = 2922)**	
**Condom use at last sex with any partner**				
	*OR (95% CI)*	Adj OR (95% CI)***	*OR (95% CI)*	Adj OR (95% CI)***
Any	1.49 (0.81, 2.74)	1.50 (0.81, 2.79)	1.44 (0.74, 2.82)	1.44 (0.73, 2.84)
Behavioral	1.56 (0.72, 3.35)	1.55 (0.72, 3.35)	1.57 (0.67, 3.66)	1.56 (0.66, 3.69)
Biomedical	1.64 (0.65, 4.13)	1.67 (0.65, 4.26)	1.73 (0.63, 4.71)	1.73 (0.62,4.81)
Complementary	1.24 (0.50, 3.05)	1.26 (0.50, 3.13)	1.11 (0.44, 2.84)	1.10 (0.42, 2.84)
Behavioral + Biomedical	2.32 (0.467, 11.46)	2.16 (0.43, 10.78)	2.48 (0.42, 14.64)	2.39 (0.40, 14.32)
Behavioral + Complementary	1.38 (0.37, 5.20)	1.33 (0.35, 5.05)	1.29 (0.33, 5.09)	1.21 (0.30, 4.88)
Combination	2.35 (0.24, 22.80)	2.26 (0.23–22.09)	2.50 (0.20, 30.82)	2.48 (0.20, 31.26)
**Condom and lubricant use at last sex with any partner**				
	*OR (95% CI)*	*Adj OR (95% CI)*	*OR (95% CI)*	*Adj OR (95% CI)*
Any	1.35 (0.90, 2.02)	1.32 (0.87, 1.99)	1.40 (0.90, 2.17)	1.31 (0.84, 2.05)
Behavioral	**1.73 (1.06, 2.83)**	**1.66 (1.01, 2.74)**	**1.84 (1.08, 3.14)**	1.70 (0.98, 2.95)
Biomedical	1.54 (0.88, 2.71)	1.53 (0.86, 2.71)	1.71 (0.94, 3.12)	1.57 (0.85, 2.90)
Complementary	0.86 (0.47, 1.58)	0.86 (0.47, 1.59)	0.86 (0.46, 1.61)	0.83 (0.44, 1.58)
Behavioral + Biomedical	**3.14 (1.22 ,8.13)**	**2.88 (1.10, 7.51)**	**3.51 (1.26, 9.80)**	**3.05 (1.08, 8.64)**
Behavioral + Complementary	1.50 (0.64, 3.54)	1.44 (0.61, 3.42)	1.56 (0.64, 3.80)	1.43 (0.58, 3.53)
Combination	3.54 (0.91, 13.75)	3.33 (0.85, 13.04)	4.12 (0.94, 18.15)	3.76 (0.84, 16.88)
**Consistent condom use in the last 30 days with all partner types**				
	*OR (95% CI)*	*Adj OR (95% CI)*	*OR (95% CI)*	*Adj OR (95% CI)*
Any	1.17 (0.66, 2.06)	1.21 (0.68, 2.16)	1.38 (0.75, 2.53)	1.37 (0.70, 2.50)
Behavioral	1.01 (0.52, 1.97)	1.05 (0.53–2.07)	1.13(0.55, 2.30)	1.17 (0.56, 2.44)
Biomedical	1.47 (0.63, 3.45)	1.45 (0.61, 3.46)	1.89 (0.75, 4.77)	1.82 (0.71, 4.65)
Complementary	0.82 (0.38, 1.80)	0.82 (0.37,1.81)	0.93 (0.41, 2.09)	0.90 (0.39, 2.04)
Behavioral + Biomedical	1.12 (0.34, 3.67)	1.09 (0.33, 3.60)	1.28 (0.37, 4.45)	1.23 (0.35, 4.37)
Behavioral + Complementary	0.71 (0.25, 2.02)	0.69 (0.24, 1.99)	0.84 (0.28, 2.48)	1.09 (0.30, 3.98)
Combination	0.72 (0.17, 3.10)	0.70 (0.16, 3.06)	0.90 (0.19, 4.22)	0.85(0.18, 4.17)
**Consistent condom use in the last 30 days with regular partners**				
	*OR (95% CI)*	*Adj OR (95% CI)*	*OR (95% CI)*	*Adj OR (95% CI)*
Any	**1.72 (1.15, 2.57)**	**1.71 (1.13, 2.57)**	**1.69 (1.09, 2.62)**	1.51 (0.88, 2.57)
Behavioral	**1.83 (1.12, 3.00)**	**1.78 (1.08, 2.93)**	**1.88 (1.10, 3.21)**	**1.88 (1.09, 3.25)**
Biomedical	1.18 (0.68, 2.05)	1.17 (0.67, 2.05)	1.19 (0.66, 2.14)	1.19 (0.65, 2.17)
Complementary	1.64 (0.90, 3.00)	1.63 (.089, 3.01)	1.50 (0.79, 2.82)	1.53 (0.80, 2.90)
Behavioral + Biomedical	1.66 (0.71,, 3.88)	1.56 (0.66, 3.67)	1.74 (0.71, 4.28)	1.63 (0.67, 4.19)
Behavioral + Complementary	1.81 (0.76, 4.30)	1.72 (0.72, 4.10)	1.69 (0.69, 4.16)	1.67 (0.67, 4.14)
Combination	1.41 (0.43, 4.54)	1.33 (0.41,4.32)	1.34 (0.39, 4.57)	1.31 (0.38, 4.56)
**Consistent condom use in the last 30 days with commercial partners**				
	*OR (95% CI)*	*Adj OR (95% CI)*	*OR (95% CI)*	*Adj OR (95% CI)*
Any	1.47 (0.91, 2.37)	1.58 (0.97, 2.59)	1.39 (0.83, 2.33)	1.51 (0.88, 2.57)
Behavioral	1.44 (0.82, 2.53)	1.58 (0.89,2.83)	1.36 (0.74, 2.50)	1.5 (0.79 ,2.78)
Biomedical	0.99 (0.51, 1.93)	1.03 (0.52, 2.03)	0.86 (0.428, 1.74)	0.9 (0.45, 1.90)
Complementary	1.87 (0.97, 3.62)	1.95 (1.00, 3.82)	1.69 (0.84, 3.38)	1.8 (0.87, 3.61)
Behavioral + Biomedical	0.88 (0.30, 2.59)	0.94 (0.32, 2.80)	0.78 (0.25, 2.44)	0.8 (0.26, 2.68)
Behavioral + Complementary	2.34 (0.96, 5.70)	**2.47 (1.00, 6.09**)	2.11 (0.84, 5.32)	2.2 (0.85, 5.69)
Combination	1.44 (0.38, 5.41)	1.52 (0.40, 5.82)	1.28 (0.312, 5.18)	1.4 (0.32, 5.66)
**Had an HIV test and received results in the last 12 months**				
	*OR (95% CI)*	*Adj OR (95% CI)*	*OR (95% CI)*	*Adj OR (95% CI)*
Any	**3.98 (2.54, 6.23)**	**4.06 (2.57, 6.42)**	**3.04 (1.88, 4.91)**	**2.98 (1.82, 4.87)**
Behavioral	**2.49 (1.49, 4.17)**	**2.48 (1.47, 4.18)**	**1.86 (1.07, 3.22)**	**1.76 (1.01, 3.10)**
Biomedical				
Complementary	**2.54 (1.28, 5.08)**	**2.55 (1.27, 5.12)**	**1.97 (1.0, 4.05)**	1.95 (0.94, 4.03)
Behavioral + Biomedical				
Behavioral + Complementary	3.52 (1.23, 10.13)	3.45 (1.19, 9.97)	2.77 (0.93, 8.30)	2.63 (0.87, 7.93)
Combination	39.58 (0.80, 1978.03)	37.78 (0.75, 1894.03)	43.22 (0.37, 5070.70)	40.03 (0.34, 4716.04)

Estimates in bold are statistically significant at p < 0.05.

*Results weighted for network size, recruitment chain, and city population size.

**Results weighted for network size, recruitment chain, city population size, and matching stratum.

***Models adjusted for age and socio-economic status.

We were able to link a total of 520 unique individuals between monitoring and survey data (Table 4). These cases were comparable to the full sample on socio-demographic factors, except that cases in Guatemala were somewhat younger on average than all men (results not shown). Consistent condom use with regular partners was higher for linked cases, except in Nicaragua. Use of condoms with water-based lubricant and HIV testing were both greater among linked cases in Costa Rica than in the full sample. Most MSM had been exposed to 1–2 behavioral activities, although in Costa Rica a quarter were exposed to more than five. Comparatively fewer of the linked cases had received any biomedical services. The country with the highest level of biomedical service use was Guatemala (22% of cases). Use of complementary services was rare. Overall, El Salvador and Nicaragua achieved the greatest levels of combination prevention exposure, but coverage was still low at 7.3% and 6.8%, respectively. No relationship between program exposure and behavioral outcomes was statistically significant when tested with chi-square tests of association.

**Table 4:** **Behavioral outcomes and program exposure for MSM cases with program monitoring and survey data, n = 520**

	Costa Rica (n = 29)	El Salvador (n = 123)	Guatemala (n = 140)	Nicaragua (n = 88)	Panama (n = 140)
**Outcomes, %**					
All partners	84.6	77.9	84.9	89.2	88.8
Regular partners	48.6	59.3	60.1	55.7	60.7
Casual partners	31	34.1	58.7	37.5	41.4
Condom use at last sex	89.7	80.3	89.6	89.8	84.1
Condom and lubricant use at last sex	65.5	30.3	59.4	43.2	64.5
Received an HIV test, past 12 months	48.3	69.9	72.5	68.2	72.1
**Combination Prevention program exposure**					
Mean number of behavioral exposures	3.35	2.41	1.8	2.43	1.44
No behavioral exposure, %	0	3.3	11.6	5.7	6.4
1–2 interactions, %	55.2	67.5	70.3	71.6	80.7
3–5 interactions, %	20.7	19.5	12.3	14.8	12.1
>5 interactions, %	24.1	9.8	5.8	8	0.7
Mean number of biomedical exposures	0.0	0.23	0.41	0.23	0.16
No biomedical exposure, %	0.0	82.9	70.3	78.4	84.3
1 biomedical service, %	0.0	13.8	22.5	20.5	15.0
> 1 biomedical service, %	0	3.3	7.2	1.1	0.7
Mean number of complementary exposures	0.04	0.08	0.01	0.08	0.07
Exposure to behavioral + biomedical, %	0.0	13.8	18.1	15.9	9.3
Exposure to behavioral + complementary, %	3.4	7.3	0.7	8	4.3
Exposure to biomedical + complementary, %	0.0	7.3	0.7	6.8	7.1
Combination prevention exposure, %	0.0	7.3	0.7	6.8	4.3

## Discussion

In light of the need for a larger evidence base on the effectiveness of combination prevention, particularly from Latin America, we have aimed in this study to systematically evaluate a program strategy that seeks to link multiple program components for a priority population in Central America. We aimed to assess population-level coverage of a combination prevention program targeted to men who have sex with men and to determine whether program exposure was associated with HIV risk reduction in this population. We present preliminary evidence here that suggests this strategy is associated with condom use with water-based lubricant at last sex, consistent condom use with regular partners, and HIV testing.

Two years into implementation, PASMO’s Combination Prevention program has achieved fairly modest levels of coverage in the target population of MSM, a population that is notably difficult to reach [54, 55]. Condom use was relatively high in this population, but condom use with water-based lubricant less so. These levels of condom use are consistent with other reports from Central America [3, 56]. Condom use in our study tended to be slightly higher, particularly with casual partners, than recent IBBS surveys in in El Salvador, Guatemala and Nicaragua [11, 12, 57]. Over half of the population had received an HIV test in the past year, except in Costa Rica where receipt of testing was less common. These results were similar to the 2008 IBBS in El Salvador, but the 2009 Nicaragua IBBS reported higher levels of HIV testing in the past year compared to our study [11, 12]. STI treatment practices were highly variable across countries, but overall trends indicate that appropriate treatment-seeking was common.

Program coverage varied across countries, indicating that the program was at different stages of penetration into the target population. Exposure to the behavioral component of the program was most common at a population level, followed closely by exposure to referral for biomedical services Panama and Costa Rica both had relatively lower levels of exposure to the behavioral component, reflecting that these countries were relatively newer sites for program implementation than Guatemala or El Salvador. Variation in biomedical program exposure was likely due in part to differing periods of program operation.

The picture of program exposure based on records of contacts from monitoring data is somewhat different. Several countries showed high service utilization, but trends differed from survey data. This suggests issues with under-reporting in the monitoring system, likely due to problems with voucher redemption, and is compounded by likely under-reporting of program exposure through recall bias in survey data. Despite the potential for discrepancies, exposure to the complementary component was consistently low across data sources and countries. The notable exception was Guatemala, where complementary exposure was 45% of exposure amongst survey respondents with any exposure. This suggests that the program was more successful in implementing the voucher mechanism and service linkages in that country.

Modalities of program implementation also account for some of the differences in exposure trends. Specifically in Costa Rica, the program operates solely through referrals to the public sector Social Security Institute, following government policy on maintaining HTC services as a public sector service. Rapid HIV tests are not yet available in Costa Rican clinics, which may have influenced outcomes there. Based on coverage levels in other countries, including in Panama where the program also only recently began implementation, loosening policy guidelines on service providers and types of HTC services that can be provided in Costa Rica may be merited to increase the use of HTC.

Despite modest levels of coverage and inconsistencies across data sources, there is evidence that participation in the Combination Prevention program was associated with HIV risk reduction. The behavioral component was associated with consistent condom use with regular partners and with using condoms and water-based lubricants at last sex. Both of these behaviors are focuses of messaging during behavioral interactions. Print materials are distributed that demonstrate the importance of using condoms with water-based lubricant, and counselors are trained to talk about lubricant use in pre- and post-counseling. Effect estimates more than doubled for men who had received both the behavioral component and referral to biomedical services, suggesting that a greater dose of exposure was particularly effective at promoting water-based lubricant use with condoms.

Based on previously collected data showing comparatively lower levels of condom use with regular partners, compared to with casual or commercial partners, PASMO specifically focused messaging within BCC activities on condom use with regular partners during the period evaluated by this study [58–61]. The finding that exposure to the behavioral component was associated with condom use with regular partners suggests that interpersonal communications and a structured approach to BCC can be effective for influencing specific behaviors. Condom use across all partner types is now relatively high, suggesting that future messaging should focus on continued maintenance of these behaviors, along with greater adoption of the use of water-based lubricants.

There was also evidence that program exposure was associated with HIV testing, particularly the exposure to the behavioral component. The effectiveness of referral to complementary services was attenuated in the matched sample, and having received both program components was associated with an even higher likelihood of HIV testing, but these results were not statistically significant, likely due to the small numbers of men who reported both components. These results suggest that BCC strategies are effectively able to promote use of HIV testing and counseling services. The program’s emphasis on improving quality of care within testing service providers, adapting service hours to be convenient for the population being served, and helping service providers to treat client respectfully likely contributed to these results, along with making referrals to these service providers. Subsequent evaluations of this program should continue to investigate whether service linkages and making services MSM-friendly are able to achieve increases in demand for and use of HTC services. This study was not designed to assess links between HIV testing and care and treatment services, but other data from the Combination Prevention program suggest that the program has contributed to improved access to care and treatment for people living with HIV [62].

After linking cases from monitoring with survey data, we found no evidence that program exposure was associated with any of the outcomes. Again, this was likely due to the rarity of exposure, a result of challenges in locating men exposed to the program during survey data collection and perhaps inconsistencies in reporting of unique identifiers. Future survey research to evaluate this program may be able to address these challenges of recall during data collection.

The Combination Prevention program results are aligned with recent evidence from the HIV prevention literature and contribute unique results on condom use with water-based lubricants, especially in regards to the doubling of effect size for men who were exposed to both the behavioral and biomedical components in the program. Several evaluations from large-scale HIV prevention efforts focused on MSM show an increase in consistent condom use with regular male partners, some with all male partners [63–65]. Community-based peer interventions show evidence of increased condom use at last sex with a male partner and increased perception of behavioral control in taking up HIV testing and counseling [66]. The Combination Prevention program, as well other programs for MSM in low and middle-income countries found to be effective, require additional investments to increase depth and breadth of program coverage.

### Limitations

This analysis has several limitations. In the survey data, recall of outcomes may have been biased. However, levels of condom use and of HIV testing were comparable to other reports from the region [56]. Program exposure recall may have been biased as well. Comparison with program monitoring data suggests that program exposure recall from survey data may have been under-reported, thus attenuating estimates of coverage and effectiveness. The survey did not collect data on HIV status, either through self-report or biomarkers, nor were we able to go beyond experience with HIV testing to look at whether this program links HIV testing and counseling to the HIV treatment cascade, priority areas for understanding the effectiveness of HIV prevention strategies and linking prevention to a continuum of care [67]. We also suspect under-reporting of program contacts in monitoring data, due to the need for program beneficiaries to redeem vouchers before registering in the system. Under-reporting may have attenuated estimates.

Another potential study limitation was in the coarsening of variables before matching. The matching procedure we used faces a trade-off in increasing the number of matched pairs at the expense of less exact matching [49, 51]. However, categorical variables were less likely to be affected by this potential source of bias. This analysis is also at risk for omitted variable bias, as we were not able to account for the effects of unobservable factors.

The measurement approach taken here primarily assesses program exposure and outcomes based on individual-level measurement. It has been argued that combination prevention programs require a multi-level exposure measurement approach or evaluation platform approach to assess the effects of structural factors [25, 26]. We were not able to measure changes at a structural level that may have been related to the program. However, measurement of the complementary component may stand in for some of these gaps, in that these services, including legal support and counseling on drug and alcohol use, influence other factors that affect risk. Further research and later rounds of the evaluation may investigate how to capture the advocacy and social mobilization components better.

## Conclusions

We have early evidence that a combination prevention strategy is associated with HIV risk reduction among MSM in Central America. Additional strategies are needed to expand program coverage into a population that is difficult to reach due to stigma and discrimination. Service linkages appear to strengthen program impacts, but measurement may be hampered by under-reporting. Future evaluations should take a multi-level approach to account for complex program operations and the multiple levels (societal, social network, interpersonal) at which a combination prevention strategy operates.

## Abbreviations

BCC: Behavior change communication
CEM: Coarsened exact matching
HIV: Human immunodeficiency virus
HTC: HIV testing and counseling
MIS: Management Information System
MSM: Men who have sex with men
PASMO: Pan-American Social Marketing Organization
PSI: Population Services International
SAM: System for applied monitoring
SES: Socioeconomic status
STI: Sexually transmitted infections
UIC: Unique identifier codes
USAID: United States Agency for International Development.
